# Antimelanogenic, Antioxidant and Antiproliferative Effects of *Antrodia camphorata* Fruiting Bodies on B16-F0 Melanoma Cells

**DOI:** 10.1371/journal.pone.0170924

**Published:** 2017-01-26

**Authors:** Jyh-Jye Wang, Chih-Chung Wu, Chun-Lin Lee, Shu-Ling Hsieh, Jin-Bor Chen, Chu-I Lee

**Affiliations:** 1 Department of Nutrition and Health Science, Fooyin University, Kaohsiung, Taiwan; 2 Department of Nutrition and Health Sciences, Chang Jung Christian University, Tainan, Taiwan; 3 Department of Life Science, National Taitung University, Taitung, Taiwan; 4 Department of Seafood Sciences, National Kaohsiung Marine University, Kaohsiung, Taiwan; 5 Division of Nephrology, Department of Internal Medicine, Kaohsiung Chang Gung Memorial Hospital and Chang Gung University College of Medicine, Kaohsiung, Taiwan; 6 Department of Medical Laboratory Science and Biotechnology, Fooyin University, Kaohsiung, Taiwan; Columbia University, UNITED STATES

## Abstract

*Antrodia camphorata* is a fungus that is endemic to Taiwan, and its fruiting body has been used as a folk medicine for the prevention or treatment of diverse diseases. The present study is aimed at investigating the antimelanogenesis and antioxidation effect of the ethanolic extract of *Antrodia camphorata* fruiting body (EE-AC), as well as its antiproliferation effects in B16-F0 melanoma cells. Regarding antimelanogenic effects, EE-AC had effective cupric ions reducing capacity and expressed more potent inhibitory effect than kojic acid on mushroom tyrosinase activity. Moreover, EE-AC significantly inhibited cellular tyrosinase activity and the melanin content in B16-F0 cells at 12.5 μg/mL concentration without cell toxicities. Regarding antioxidant effects, EE-AC exhibited potent DPPH radical- and SOD-like-scavenging activities. Regarding antiproliferative effects, EE-AC exhibited a selective cytotoxic effect and markedly inhibited the migration ability of B16-F0 cells. EE-AC increased the population of B16-F0 cells at sub-G1 phase of the cell cycle. EE-AC also caused the increase of early apoptotic cells and chromatin condensation, which indicated the apoptotic effects in B16-F0 cells. We demonstrated that EE-AC possessed antimelanogenic, antioxidant and anti-skin cancer actions. The results would contribute to the development and application of cosmetics, healthy food and pharmaceuticals.

## Introduction

*Antrodia camphorata* (synonym *Antrodia cinnamomea*) is a highly valued mushroom that is endemic to Taiwan. It is rare and cannot be cultivated because it grows only on the inner heartwood wall of the endemic evergreen *Cinnamonum kanehirai* [1]. The fruiting body of *A*. *camphorata* has been traditionally used as a medicine to treat food intoxication and liver disease by the aboriginal Taiwanese [2]. *A*. *camphorata* crude extract has been proven to possess numerous healthy characteristics and functional metabolites [3]. However, the physiologically active ingredients of *A*. *camphorata* are yet to be identified.

Melanoma is a highly aggressive and invasive skin cancer, and occurs most commonly among the Caucasian. It is usually caused by skin cell damage resulting from exposure to ultraviolet light from the sun. Melanogenesis is a physiological process that results in the synthesis of melanin pigments, and tyrosinase is a key enzyme for melanin biosynthesis [4]. Melanin production is also related to intracellular free radicals generation and antioxidant (i.e., glutathione) level [5]. Synthesis of melanin pigment also plays a crucial role in protecting humans from skin cancer. The formation of melanin precipitate caused by ultraviolet light could be prevented by adding a tyrosinase inhibitor and antioxidant compound to cosmetic or skin care products. Existing whitening cosmetic products include ingredients such as ascorbic acid, kojic acid, arbutin, and hydroquinone [6]. However, the development of safer and more effective whitening agents is still needed.

Regarding antioxidant effects, the extracts from *A*. *camphorata* mycelia have been found to be free radical scavengers in possessing antioxidant activity [7]. However, up to now, there has been little work carried out on the antioxidant effects of *A*. *camphorata* fruiting bodies. The anti-tumor physiological effects of *A*. *camphorata* fruiting bodies have been reported in various cancers *in vitro* and *in vivo* [8–10]. Recently, one report showed that a fermented culture broth of *A*. *camphorata* could inhibit melanoma proliferation and progression [11]. We previously reported that the functional compounds including triterpenoids, polysaccharides and 1,3-β-D-glucan derived from the fruiting body of *A*. *camphorata* exert anticancer effects on human pancreatic cancer cells [12]. However, the therapeutic potential of natural *A*. *camphorata* fruiting body to kill melanoma cells and the main mechanisms that underlie its anticancer effect need to be further investigated.

Since the traditional medicinal uses of *A*. *camphorata* are not fully investigated and its other effects have not been clarified, as part of our continued search for novel bioactive natural food, we investigated the antimelanogenesis, antioxidation effect of the ethanolic extract of *A*. *camphorata* fruiting body (EE-AC), as well as its antiproliferation effects in B16-F0 mouse melanoma cells. This study is the first to explore new applications of *A*. *camphorata*, which provides valuable information about the development of potential depigmenting agents such as skin-whitening cosmetics and a multifunctional healthy food for skin cancer prevention.

## Materials and Methods

### Chemicals and reagents

Annexin V-FITC apoptosis detection kits were purchased from Strong Biotech (Taipei, Taiwan). Cisplatin, MTT, PI and other chemicals which not specified above were purchased from Sigma Chemical Co. (St. Louis, MO, USA).

### Preparation of the ethanolic extract from *A*. *camphorata* fruiting body (EE-AC)

The fruiting body of *A*. *camphorata* (grown on the root of wild *C*. *kanehirai* Hay) was provided by Sky Tree Biotechnology Co., Ltd (Kaohsiung, Taiwan); it was collected from mountain areas in southern Taiwan. Preparation of the EE-AC was performed as previously described [12]. Briefly, for the preparation of the ethanolic extract, dried fruiting bodies samples were ground and then the powdered fruiting bodies were combined with 95% ethanol in a 1:20 (w/v) ratio and shaken for 24 h at room temperature. The supernatant was filtered and then centrifuged at 3000×*g* for 30 min. Later, a rotary evaporator under vacuum was used for the lyophilization process of the ethanol extracts.

### Total polysaccharides analysis

The concentration of polysaccharides was measured according to the procedures of a previously published method by the phenol-sulfuric acid assay using glucose as a standard [12]. The extracted sample was precipitated using 95% ethanol at 4°C for 24 h. The precipitated polysaccharides were centrifuged at 3000×*g* for 15 min, dried and dissolved with distilled water. A reading of 490 nm in a spectrophotometer was recorded.

### Total 1,3-β-D- glucan analysis

Measurement of the total (1→3)-β-D-glucan concentration was performed according to the procedures of a previously published method by the aniline blue assay [12]. Briefly, the sample was dissolved using 0.3 N NaOH at 25°C for 30 min. Then the sample aliquot was reacted with aniline blue at 25°C for 2 h. The fluorescence was assayed using a fluorescence spectrophotometer with excitation and emission wavelengths of 395 and 495 nm, respectively.

### HPLC analysis of total triterpenoids

Qualitative analysis of total triterpenoids was performed according to the procedures of a previously published method [12]. Briefly, 0.1 mg of the *A*. *camphorata* fruiting body was dissolved in 10 mL of 100% methanol and then filtered through a 0.22 μm membrane to prepare the sample solution. The 10 μL sample solution was analyzed using HPLC (Model L-2130, Hitachi, Japan). The detection of the absorbance using a photodiode array detector at 275 nm was carried out. The model of the analytical column was Intertsil ODS-3 C-18 C18 column, 25 cm × 4.6 mm i.d., 5 μm. The mobile solution eluded, containing of 0.2% formic acid and 100% acetonitrile at a flow rate of 1.0 mL per min was performed. A colorimetric method was used to determine the total triterpenoid content of *A*. *camphorata* fruiting bodies. Briefly, under reflux in 5 mL of 95% ethanol for 30 min, the extraction of 0.5 g sample was gained. The extraction was then filtered and centrifuged at 3000×*g* for 10 min. One mL of the diluted solution (1/10) was dried, and 0.2 mL of 5% (w/v) vanillin–glacial acetic acid and 0.08 mL of perchloric acid were added sequentially after the extraction was done. The mixture was shaken in a 60°C water bath for 20 min. After being added to 3.72 mL of glacial acetic acid against a standard oleanolic acid curve, a 550 nm absorbance (UV visible spectrophotometer, Model U-2800, Hitachi, Japan) was used to read the concentration of total triterpenoids.

### Determination of total phenolic compounds

The concentration of total phenolic compounds was measured according to the procedures of a previously published method [13]. Regarding the determination of total phenolic compounds, EE-AC was dissolved in deionized water, and the concentration of the compounds was quantitatively analyzed using Folin–Ciocalteu’s reagent spectrophotometrically. Sample solution (0.5 mL), Folin–Ciocalteu’s reagent (0.5 mL), sodium carbonate (1.0 mL, 75 g/L), and deionized water (2.5 mL) were all thoroughly mixed and kept at 25°C for 30 min, the absorbance was later read at 765 nm. Determination of total phenolic content was carried out using gallic acid as standard.

### Determination of total flavonoids

The concentration of total flavonoids was measured according to the procedures of a previously published method [13]. For measuring the content of flavonoids, sample solution (0.5 mL), water (2.0 mL), 5% NaNO_2_, and 4% NaOH (2.0 mL) were well mixed and kept at room temperature for 15 min. Next, a spectrophotometer at 415 nm was used to measure the absorbance. Lastly, calculation of the total flavonoids content was determined based on an established standard curve of rutin.

### Cupric ions (Cu^2+^) reducing power

Measurement of Cu^2+^ reducing power was performed according to a previously published method [14]. Briefly, a volume of 0.2 mL of EE-AC was added to 0.4 mL of CuSO_4_ aqueous solution (0.4 mM) mixed vigorously, and incubated for 10 min. Then, a 0.4 mL of bathocuprinedisulfonic acid (4 mM) was added to the mixture. Absorbance against a buffer blank was measured at 483 nm after 10 min.

### Mushroom tyrosinase inhibition assay

Measurement of mushroom tyrosianse inhibition was performed as described previously with slight modifications [15]. A volume of 25 μL of 0.5 mM L-DOPA solution, 25 μL of 10 mM L-tyrosine, 875 μL of 50 mM phosphate buffer (pH 6.5) and 25 μL of the EE-AC solution were mixed. Then, 50 μL of mushroom tyrosinase (1600 U/mL) was added and mixed well. After incubation for 30 min, the absorbance at 475 nm was measured with a microplate reader.

### Cell culture

B16-F0 (Murine melanoma cells; ATCC Number: CRL-6322^™^) and human embryonic kidney cells (HEK-293; ATCC CRL-157) were cultured with DMEM supplemented with 10% fetal bovine serum with the mixture of 100 IU/mL penicillin and 100 μg/mL streptomycin at 37°C in a 5% CO_2_ humidified incubator.

### Measurement of intracellular tyrosinase activity

Measurement of intracellular tyrosinase activity was performed according to a previously published method with slight modifications [16]. In brief, cells (2.5×10^4^ cells/0.5 mL of medium containing EE-AC) were plated in each well of a 24-well plate and incubated for 48 h. Then, the cells were lysed with 1% Triton-X by freeze-thawing. The lysate was centrifuged at 16000×g for 20 min. Finally, 100 μL of each supernatant was added to 100 μL of 2 mM L-DOPA and absorbance was measured at 492 nm in a microplate reader.

### Measurement of melanin content

Cells (2.5×10^4^ cells/0.5 mL of medium containing EE-AC) were plated in each well of a 24-well plate and incubated for 48 h. Then, the cells were dissolved in 100 μL of 1N NaOH after twice washing with PBS and boiled for 30 min to solubilize the melanin. The lysate was centrifuged at 16000×*g* for 20 min. Then, the absorbance at 405 nm of the supernatant was measured in a microplate reader.

### DPPH radical-scavenging activity assay

The reaction mixture contained 1mL of 100 μM DPPH in 1 mL ethanol and 50 μL of sample solution in 0.95 mL Tris-HCl buffer (0.05 M, pH 7.4). After the reaction was carried out at room temperature for 30 min, the free-radical-scavenging activity of the sample was quantified by the decolorization of DPPH at 517 nm.

### SOD-like-scavenging activity assay

One milliliter of 120 μM PMS, 1mL of 936 μM NADH and 1 mL of 300 μM NBT were added to 1.0 mL sample solution sequentially. After the reaction was carried out at room temperature for 5 min, the superoxide anion-scavenging activity of the sample was quantified by the decolorization of SOD-like at 560 nm.

### Measurement of cell viability

Cell viability assay was performed as previously described [12]. Briefly, cells (5×10^3^ cell) were plated in each well of a 96-well plate. After cells were exposed to various concentrations of EE-AC for 48 h, a 50 μL MTT solution (5 mg/mL in stock solution) was added to each well. After the cells were incubated at 37°C for 2 h, formazan crystals were formed and dissolved with DMSO (100 μL). Next, an ELISA reader was used to read the absorbance at 570 nm.

### Wound healing assay

Wound healing assay was performed as previously described [12]. Briefly, the cells were cultured abundantly in petri dishes. After treatment, using a 200 μL pipette tip, the cell monolayer was scratched in shape of a line. The cells were photographed (100× magnification) at scheduled time periods to monitor the movement of cells into the wounded area. Finally, calculation of the closure of wounded area was being recorded.

### Cell cycle analysis

Cell cycle analysis was performed as previously described [12]. Briefly, fixation of the cells was carried out with 70% ethanol at -4°C for 18 h. Then, the staining solution [1 mg/mL PI] was added to the cells and incubated at 25°C for 30 min. Later, FACScan flow cytometer combined with ModFit LT V3.0 software were used to analyze the cell cycle.

### Annexin V-FITC/PI doubling staining

According to the manufacturer’s operation manual, the cells were stained with Annexin V-FITC and PI. A FACScan flow cytometer was used to analyze each sample of at least 10,000 cells after the cells were incubated in the dark at 25°C for 15 min.

### Hoechst 33258 staining assay

Examination of the nuclear morphology was performed as previously described [12]. Briefly, fixation of the cell is carried out using 4% paraformaldehyde at 4°C for 30 min. The cells were then incubated in nuclear fluorochrome Hoechst 33258 at a final concentration of 5 μg/mL at room temperature for 30 min. Finally, an inverted fluorescence microscope was used to examine the chromatin condensation.

### Statistics

Data are presented as means ± SD and analyzed using Student's *t*-test. A *P* value of less than 0.05 was considered as statistically significant.

## Results

### Functional metabolites of the *A*. *camphorata* fruiting body

The sample studied in the present work was obtained by extraction of the total ethanol fraction of the *Antrodia camphorata* fruiting body. The yields in weight percentage of residues, referring to the weight of dry material extracted, were 3.59% of EE-AC (w/w). The EE-AC contains certain levels of bioactive compositions, including total polysaccharides, 1,3-β-D-glucan, triterpenoids, polyphenols and flavonoid. The total polysaccharide content in EE-AC was 202.35 ± 15.24 mg/g. The total 1,3-β-D-glucan content in EE-AC was 81.34 ± 3.89 mg/g. The total triterpenoid content in the *A*. *camphorata* fruiting body was determined using oleanolic acid as the reference standard to be 5.62 ± 0.64%. The total polyphenol content of EE-AC was 78.56 ± 6.86 mg of gallic acid equivalent/g of extract. The flavonoid production of EE-AC was 11.52 ± 1.85 mg of rutin equivalent/g of extract (Table 1).

**Table 1:** Total polysaccharides, 1,3-β-D-glucan, triterpenoids, polyphenols, and flavonoids of *A*. *camphorata* fruiting body.

Polysaccharides (mg/g)	1,3-β-D-glucan (mg/g)	Triterpenoids (%)	Polyphenols^a^ (mg/g)	Flavonoids^b^ (mg/g)
202.35 ± 15.24	81.34 ± 3.89	5.62 ± 0.64	78.56 ± 6.86	11.52 ± 1.85

^*a*^gallic acid equivalent.

^*b*^Rutin equivalent.

Values are presented as the means ± SD (n = 3).

### Antimelanogenesis of EE-AC

**Fig 1:**
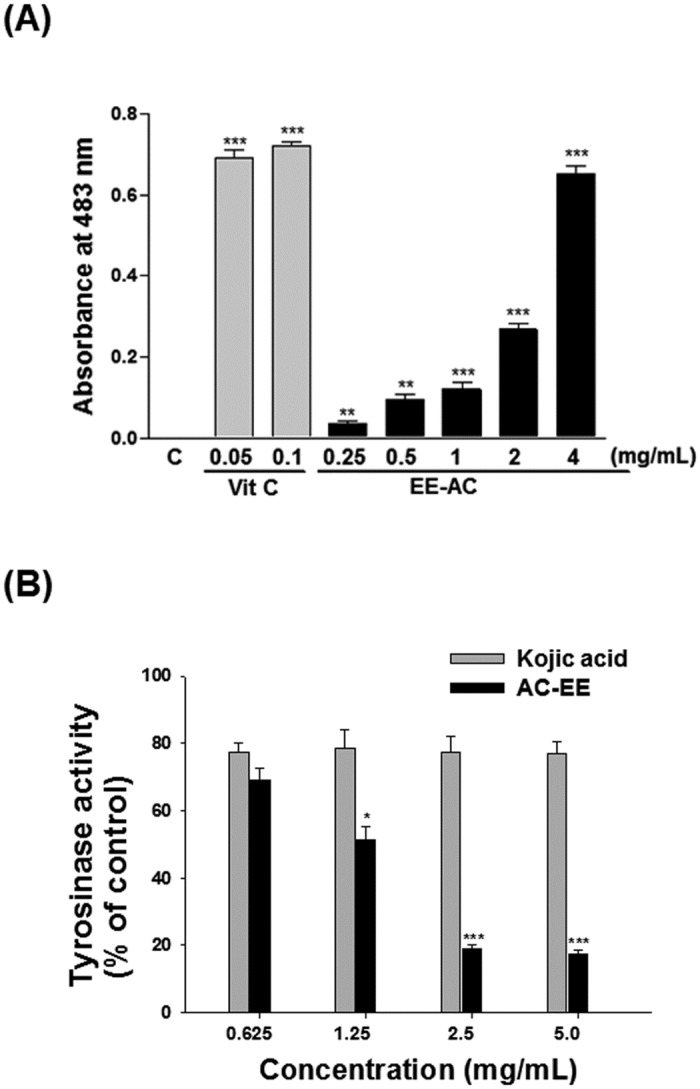
Anti-melanogenic effect of EE-AC in cell-free system. (A) Determination of Cu^+2^ reducing power of EE-AC. Vitamin C (Vit C) was used as a reference antioxidant. Values are significantly different by comparison with the negative control (C; 0.1% DMSO), and the data are presented as mean ± SD. **p < 0.01; ***p < 0.001. (B) Determination of mushroom tyrosinase activity. Kojic acid was used as a positive control. Results are represented as percentages of negative control (0.1% DMSO), and the data are presented as mean ± SD. Values are significantly different by comparison with the negative control. *p < 0.05; **p < 0.01; ***p < 0.001.

**Fig 2:**
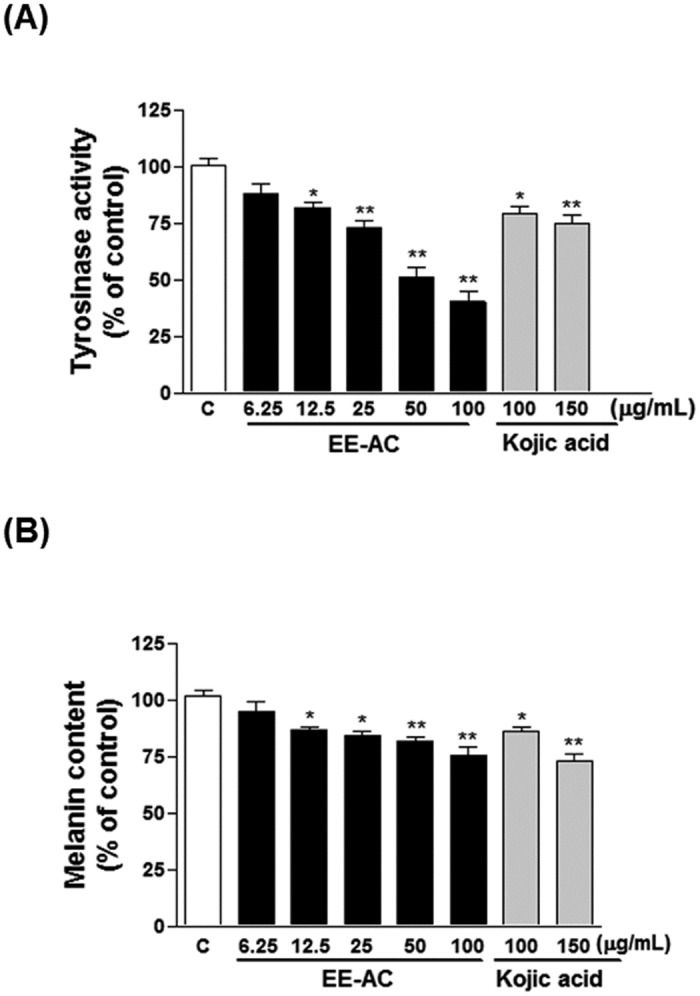
Anti-melanogenic effect of EE-AC in B16-F0 melanoma cells. (A) Effect of EE-AC on cellular tyrosinase activity in B16-F0 cells. The cells were exposed to increasing doses of EE-AC for 48 h. Kojic acid was used as a positive control. (B) Effect of EE-AC on cellular melanin content in B16-F0 cells. The cells were exposed to increasing doses of EE-AC for 48 h. Kojic acid was used as a positive control. Results are represented as percentages of negative control (0.1% DMSO), and the data are presented as mean ± SD. Values are significantly different by comparison with the negative control. *p < 0.05; **p < 0.01.

To elaborate the potential of EE-AC for usage in skin-whitening cosmetics, we demonstrated their antimelanogenic actions. Since tyrosinase is the key enzyme in the melanin biosynthesis, and Cu^2 +^ was a cofactor, which has oxygenase and oxidase dual function, we first measured the Cu^2+^ reducing ability of EE-AC on antimelanogenesis effect. As shown in Fig 1A, EE-AC exhibited effective Cu^2+^ reducing capacity in a concentration-dependent manner. The Cu^2+^ reducing capacity at 4 mg/mL concentration of EE-AC showed comparative reducing ability to 0.05 mg/mL of vitamin C. By using the colorimetric method to detect the inhibitory effects on mushroom tyrosinase, EE-AC groups expressed more potent inhibitory effect on tyrosinase activity than kojic acid (the positive control). The IC_50_ value for EE-AC on the mushroom tyrosinase activity was found as 1.25 mg/mL (Fig 1B). A lower IC_50_ value indicates a higher inhibitory effect on tyrosinase activity. Therefore, the antimelanogenic effects of EE-AC were attributable to their inhibitory effects on tyrosinase via their Cu^2+^ reducing action. In Fig 2A, EE-AC demonstrated noticeable superior inhibition at a concentration-dependent manner to cellular tyrosinase activity in B16-F0 cells. The IC_50_ value for EE-AC on the cellular tyrosinase activity was found as 50 μg/mL. As shown in Fig 2B, EE-AC treatments caused dramatic decreases in the melanin content in melanoma cells in a dose-dependent manner. The melanin contents matched with the tyrosinase activities in the same dose-dependent tendencies upon EE-AC, indicating that the cellular melanin decreases might be due to the inhibition of tyrosinase activities. EE-AC inhibited cellular tyrosinase activity and melanin production as effectively as kojic acid, one of the most widely used inhibitor of the formation of pigment in cosmetics used for skin brightening. These results imply that EE-AC displayed hypopigmenting action, making it a good candidate for skin-whitening materials.

### Antioxidant activity of EE-AC

**Fig 3:**
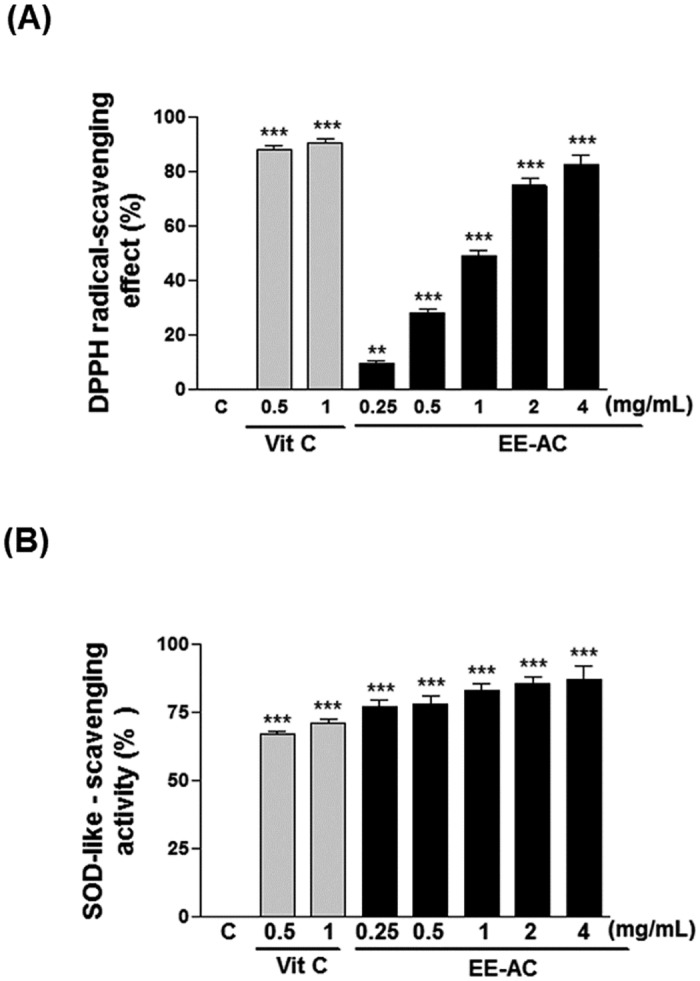
Antioxidant effects of EE-AC. (A) Determination of DPPH-scavenging ability. (B) Measurement of SOD-like-scavenging activity. Vitamin C (Vit C) was used as a positive control. Values are represented as percentage of negative control (C; 0.1% DMSO). Data are presented as mean ± SD. **p < 0.01; ***p < 0.001.

The DPPH radical scavenging activity of EE-AC was examined by comparing it with the activities of a known antioxidant such as vitamin C. The results are shown in Fig 3A. 4 mg/mL of EE-AC was highly effective with approximate 82.98% activity, which was almost as great as the 88.12% activity of vitamin C (0.5 mg/mL). As shown in Fig 3B, compared to vitamin C (approximate 66.92% at 0.5 mg/mL), EE-AC exhibited a higher superoxide anion-scavenging activity at indicate concentrations (the SOD-like activities of the EE were approximately 71.17%〜86.86% at 0.25〜4 mg/ml concentration). It is considered that EE-AC acted as a direct free radical scavenger in the antioxidant activity.

### Antiproliferative effects of EE-AC

**Fig 4:**
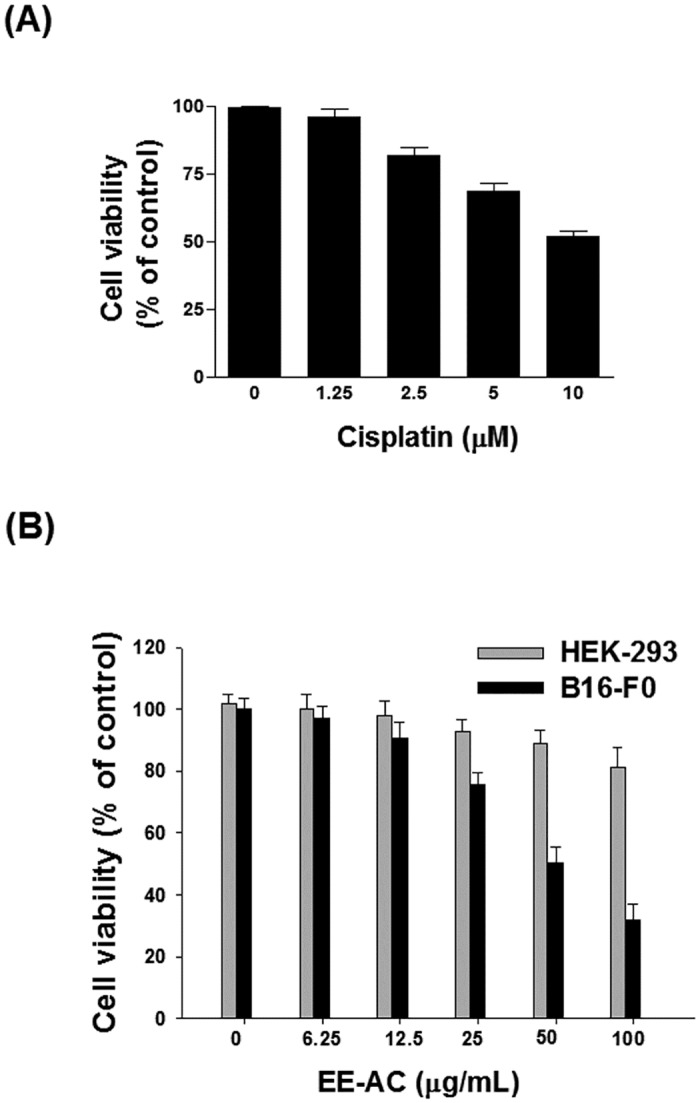
The cytotoxic effect of EE-AC on B16-F0 cells. Cells were treated with 0.1% DMSO (negative control) or various concentrations of drug for 48 h. Cell viability was measured by MTT assay. (A) Cytotoxicity of cisplatin against B16-F0 cells. (B) Cytotoxicity of EE-AC against B16-F0 cells (Black bars) or HEK-293 cells (Grey bars).

We examined the effect of EE-AC on the viability of B16-F0 cells using MTT assay. As shown in Fig 4A and 4B, treatment for 48 h with cisplatin or EE, both showed dose-dependent inhibition on the survival of the B16-F0 cells. In the positive control groups, IC_50_ (μg/mL) of cisplatin at 48 h was 10 μM. In the tested groups, EE-AC was found to be toxic to B16-F0 cells with IC_50_ values of 50 μg/mL, and posed no significant toxicity to human normal HEK-293 cells at the same concentration. It is worth noting that EE-AC was proved to have good cellular inhibition of tyrosinase activity and melanin production at 12.5 μg/mL, and had no cytotoxicity effect to human normal cells.

**Fig 5:**
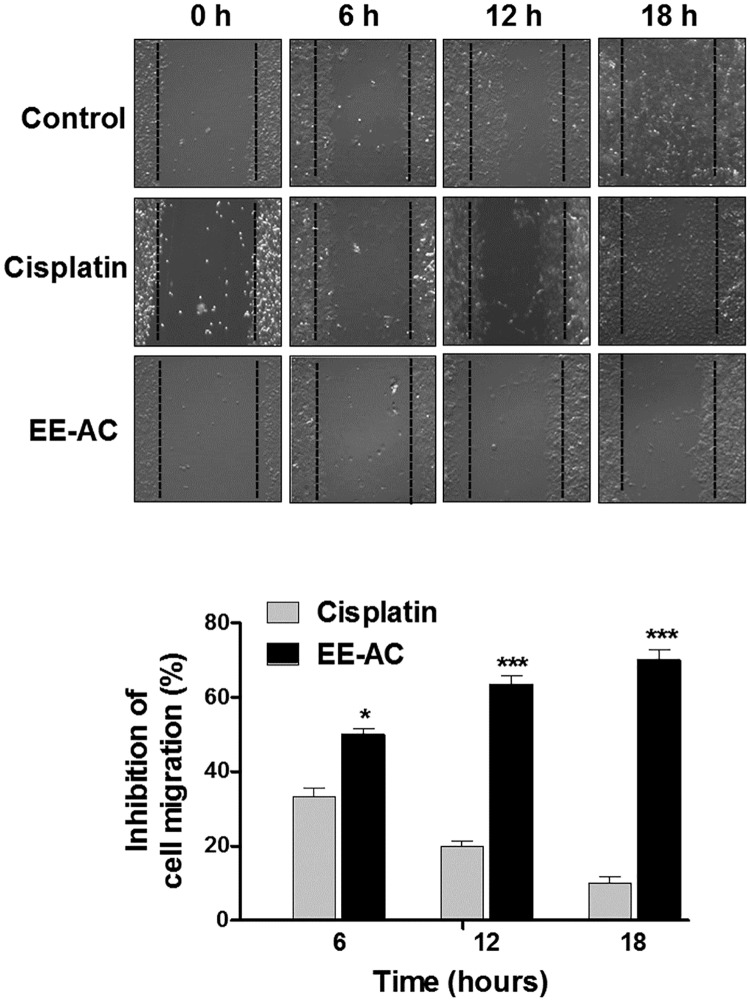
EE-AC inhibits the motility of B16-F0 cells. B16-F0 cells were scratched and treated with 0.1% DMSO (negative control) or IC_50_ values of cisplatin (10 μM) or EE-AC (50 μg/mL). Inhibition of migration was observed using a phase contrast microscope (100 × magnification) at 0, 6, 12 and 18 h (Top panel), and the closure of the wound area was calculated (bottom panel). Values are significantly different by comparison with the cisplatin group. *p < 0.05; ***p < 0.001.

Melanoma is a highly malignant tumor with a high metastatic rate. Hence, wound healing assay was performed to evaluate cancer cell migration ability. We compared EE-AC with the negative control group (0.1% DMSO) and the positive control group (10 μM cisplatin). The results demonstrated that EE-AC significantly suppressed the migration of B16-F0 cells in a time-dependent manner, and its inhibitory effect was even more significant than that of cisplatin, a common chemotherapeutic agent for solid malignancies (Fig 5).

The decreased cell viability could be the result of the inhibition of growth and/or the induction of apoptosis. We therefore investigated whether EE-AC could induce cell cycle arrest and apoptosis in melanoma cells. To examine the effect of EE-AC on cell cycle regulation in B16-F0 cells, the ploidy state of cells was monitored by flow cytometry after PI staining nuclei. As shown in Table 2, compared with the vehicle-treated controls, EE-AC increased the population of cells at sub-G1 phase of the cell cycle. After 48 h of EE-AC treatment, the sub-G1 population increased to ∼4.03%. Concurrently, the cisplatin group caused a relatively lower increase of the sub-G1 population (to ∼1.13%).

**Table 2:** Effects of cisplatin and the ethanolic extract of *Antrodia camphorata* fruiting body (EE-AC) on cell cycle regulation in B16-F0 cells.

Phase	Treatment		
	Control (0.1% DMSO)	Cisplatin (10 μM)	EE-AC (50 μg/mL)
Sub-G1	0.06 ± 0.01%	1.13± 0.09%*	4.03 ± 0.23%**
G0/G1	57.6 ± 1.74%	58.2 ± 2.55%	49.4% ± 3.19%
S	31.2 ± 1.53%	31.5 ± 2.02%	34.5 ± 1.97%
G2/M	10.2 ± 0.37%	9.3 ± 0.26%	12.1 ± 0.88%

Cell cycle analysis of B16-F0 cells after treatment with DMSO (0.1%), cisplatin and EE-AC (IC_50_) for 48 h. After drug treatment, cells were examined with PI fluorescence and analyzed by flow cytometry. The data were normalized and calculated as a percentage of the control value and they represent the mean ± SD.

Significantly different from the control group at

*p<0.05,

**p<0.01.

**Fig 6:**
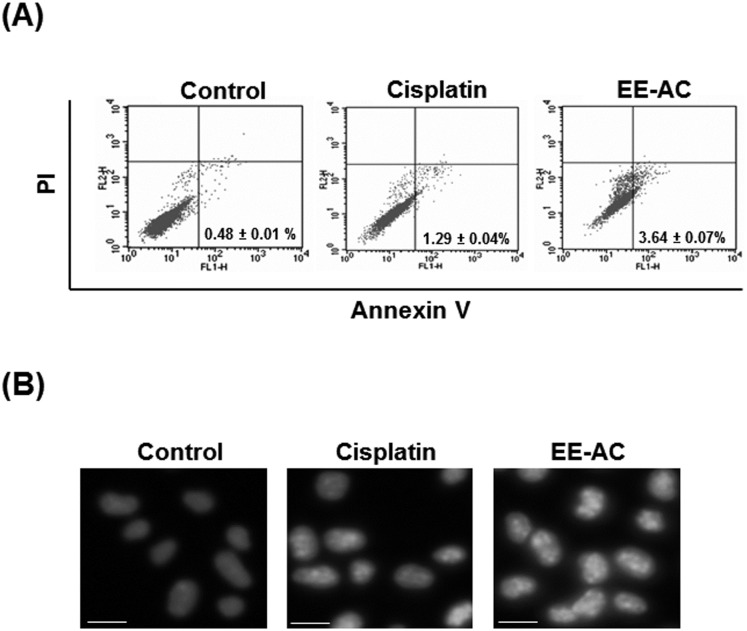
Effects of EE-AC on the induction of apoptosis in B16-F0 cells. (A) Cells were treated with 0.1% DMSO (negative control) or IC_50_ values of cisplatin (10 μM) or EE-AC (50 μg/mL) for 48 h and then stained with annexin V-FITC and PI. The annexin V-FITC signal is shown on the X axis; the PI signal is shown on the Y axis. A representative dot plot of the FACScan profile shows the percentage of early apoptotic cells in the right-bottom panel of each plot. (B) Cells were treated with 0.1% DMSO (negative control) or cisplatin (10 μM), or EE-AC (50 μg/mL) for 48 h. Nuclear morphology was examined with an inverted fluorescence microscope. Arrows indicate condensed or fragmented nuclei. Scale bars represent 10 μm.

To prove that EE-AC inhibited B16-F0 cell proliferation by induction of apoptosis, the percentage of apoptotic cells was examined on phosphatidylserine externalization in B16-F0 cells and flow cytometric analysis. As shown in Fig 6A, an obvious level of early apoptosis (annexin V-FITC positive and PI negative) was observed in EE-AC-treated B16-F0 cells (∼3.64%), whereas a much lower level of apoptosis was observed in cisplatin-treated B16-F0 cells (∼1.29%). The apoptotic cells were further evidenced by changes in nuclear morphology. In intact control cells, cells presented nuclei with homogenous chromatin distribution (Fig 6B). However, the chromatin condensation was observed in both EE-AC and cisplatin-treated B16-F0 cells, and this proapoptosis effect of EE-AC was more significant than that of cisplatin.

## Discussion

We found a new natural resource, EE-AC that shows high inhibitory activities against melanogenesis. Kojic acid has been shown to inhibit tyrosinase from various sources including fungi, plants, and animal cells; its depigmenting action is attributed to an ability to chelate copper at the active site of tyrosinase that may lead to the loss of tyrosinase activity [17]. *In vitro* testing revealed that EE-AC possessed great efficacy of tyrosinase inhibitory activity as evident by its Cu^2+^ reducing ability. Therefore, EE-AC is also a good chelator of copper. One of the interesting findings of our study is that although AC-EE is not a pure compound, it exerts comparable antimelanogenesis activity as kojic acid, which suggests that it should have the potential to be developed into a whitening agent. Because safety is a major concern when developing a potential whitening agent, cell viability assay was performed in B16-F0 melanoma cells and HEK-293 human normal cells to assess the cell safety of EE-AC. We found that EE-AC at a concentration of 12.5 μg/mL has a strong antimelanogenic effect on B16-F0 cells but does not show any cytotoxic effect on B16-F0 and HEK-293 cells. This is the first report on the antimelanogenesis effect of *A*. *camphorata*, and the results showed that EE-AC may be superior to kojic acid as a whitening agent because of its natural source, predominant bioactive compounds and safety. Further investigations are required to verify the antimelanogenic effect of EE-AC in an animal model.

Melanoma is a highly aggressive skin cancer due to its metastasizing potential and resistance to conventional cancer therapies. Cell migration is essential for cancer cell invasion and metastasis. In this study, the migration rate of B16-F0 cells was markedly inhibited by exposure to EE-AC, and this inhibitory migration effect was more significant than that of cisplatin, a common chemotherapy drug for various types of cancers. We also demonstrated that the decrease in cell viability by EE-AC was due to the induction of cells with a sub-diploid DNA content, and apoptosis in B16-F0 cells. The proapoptosis effect induced by EE-AC was evidenced as the increase of apoptotic cells by FITC-conjugated annexin V and chromatin condensation measurement, indicating that EE-AC-induced cytotoxicity was involved in the apoptosis mechanism. In addition, this proapoptosis effect of EE-AC was more significant than that of cisplatin. The inhibition of migration and viability results imply that EE-AC is a potential anticancer agent for skin cancer. The results may provide new insight and deepen the understanding of the chemopreventive properties of EE-AC. Moreover, these results offer the possibility of developing the *A*. *camphorata* fruiting body into healthy food supplements for effective chemopreventive treatment for skin cancer. Nevertheless, further research on the effectiveness of EE-AC treatment in melanoma cells is still necessary. It has been reported that some fungal and plant extracts, which are rich in bioactive metabolites, are beneficial for melanoma prevention and therapy [18–20]. In the present study, our data showed that EE-AC demonstrated notable cytotoxic effects on B16-F0 cells, which is comparable to the potent anticancer activities of those fungal and plant extracts on melanoma cells.

In the present study, the results demonstrated that like vitamin C, EE-AC also possesses potent DPPH radical and SOD-like scavenging activities, which verifies previously reported data revealing antioxidant activity and showing as a free radical scavenger of *A*. *camphorata* [7]. Polysaccharides of *A*. *camphorata* have been found to be an edible free radical scavenger in preventing oxidative damage in living organism [21,22]. Recent data also suggested that β-D-glucans exhibit strong activity of scavenging free radicals and have been explored as novel potential antioxidants [23]. Recently, polysaccharides and β-D-glucans existing *A*. *camphorata* are also related to anticancer properties [12]. In addition, triterpenoids are considered as one of the most biologically functional compounds for antioxidant and antiproliferative actions in extracts of *A*. *camphorata* [7,24,12]. Polyphenols and flavonoids derived from *A*. *camphorata* also possessed potential applications in antioxidant activity and antiproliferative properties [22]. Apart from exhibiting antioxidant and antiproliferative activities in various natural food products, polyphenols and flavonoids are scavengers of ROS production that can reduce hyperpigmentation and prevent UV-induced melanogenesis [6,25]. Therefore, polyphenols and flavonoids are considered the potential sources of skin-whitening agents. In the present study, the functional metabolites identified from the *A*. *camphorata* fruiting body are predominantly polysaccharides, 1,3-β-D-glucan, triterpenoids, polyphenols and flavonoid; this suggests that these five functional components of *A*. *camphorata* should contribute to the regulation of melanoma cell melanogenesis, oxidation and growth. However, the specific effect of each bioactive ingredient of *A*. *camphorata* need further study.

In sum, the present study demonstrated, for the first time, the potential effects of EE-AC have antimelanogenetic, antioxidant and antiproliferative activities in melanoma cells. These results suggested that the *A*. *camphorata* fruiting body could be an effective substance in skin-whitening cosmetics and healthy food supplements, as well as a chemopreventive agent against human skin cancer.

## Abbreviations

DMEM: Dulbecco's Modified Eagle Medium
DMSO: Dimethyl sulfoxide
DPPH: 1,1-Diphenyl-2-picrylhydrazyl
EE-AC: ethanolic extract of *Antrodia camphorata*
ELISA: enzyme-linked immunosorbent assay
FITC: fluorescein isothiocyanate
HPLC: high-pressure liquid chromatography
L-DOPA: L-3,4-dihydroxyphenylalanine
MTT: 3-(4,5-dimethylthiazol-2-yl)-2,5-diphenyltetrazolium bromide
NADH: dihydronicotineamidadenine dinucleotide
NBT: nitroblue tetrazolium
PI: propidium iodide
PMS: phenazine methosulphate
ROS: reactive oxygen species
SD: standard deviation
SOD: superoxide dismutase
